# Odorant receptor co-receptors affect expression of tuning receptors in *Drosophila*

**DOI:** 10.3389/fncel.2024.1390557

**Published:** 2024-05-20

**Authors:** Teng Long, Pratyajit Mohapatra, Sydney Ballou, Karen Menuz

**Affiliations:** ^1^Department of Physiology and Neurobiology, University of Connecticut, Storrs, CT, United States; ^2^Connecticut Institute for the Brain and Cognitive Sciences, University of Connecticut, Storrs, CT, United States; ^3^Institute for Systems Genomics, University of Connecticut, Storrs, CT, United States

**Keywords:** olfaction, receptor, transcriptome, *Drosophila*, olfactory sensory neuron, antenna

## Abstract

Insects detect odorants using two large families of heteromeric receptors, the Odorant Receptors (ORs) and Ionotropic Receptors (IRs). Most OR and IR genes encode odorant-binding “tuning” subunits, whereas four (*Orco*, *Ir8a*, *Ir25a*, and *Ir76b*) encode co-receptor subunits required for receptor function. Olfactory neurons are thought to degenerate in the absence of *Orco* in ants and bees, and limited data suggest this may happen to some olfactory neurons in *Drosophila* fruit flies as well. Here, we thoroughly examined the role of co-receptors on olfactory neuron survival in *Drosophila*. Leveraging knowledge that olfactory neuron classes are defined by the expression of different tuning receptors, we used tuning receptor expression in antennal transcriptomes as a proxy for the survival of distinct olfactory neuron classes. Consistent with olfactory neuron degeneration, expression of many OR-family tuning receptors is decreased in *Orco* mutants relative to controls, and transcript loss is progressive with age. The effects of Orco are highly receptor-dependent, with expression of some receptor transcripts nearly eliminated and others unaffected. Surprisingly, further studies revealed that olfactory neuron classes with reduced tuning receptor expression generally survive in *Orco* mutant flies. Furthermore, there is little apoptosis or neuronal loss in the antenna of these flies. We went on to investigate the effects of IR family co-receptor mutants using similar approaches and found that expression of IR tuning receptors is decreased in the absence of *Ir8a* and *Ir25a*, but not *Ir76b*. As in *Orco* mutants, Ir8a-dependent olfactory neurons mostly endure despite near-absent expression of associated tuning receptors. Finally, we used differential expression analysis to identify other antennal genes whose expression is changed in IR and OR co-receptor mutants. Taken together, our data indicate that odorant co-receptors are necessary for maintaining expression of many tuning receptors at the mRNA level. Further, most *Drosophila* olfactory neurons persist in OR and IR co-receptor mutants, suggesting that the impact of co-receptors on neuronal survival may vary across insect species.

## Introduction

Insects use olfaction to navigate their environments, contributing to recognition of food, toxins, and mates. Odorants are detected by their peripheral olfactory organs, the antennae and maxillary palps. Each appendage is densely covered in cuticular hairs known as sensilla, which encompass the dendrites of olfactory neurons. In *Drosophila melanogaster*, the antenna and maxillary palp have ∼1,200 and ∼120 olfactory neurons, respectively, with 1–4 olfactory neurons per sensillum (Stocker, 1994). The ∼45 olfactory neuron classes in *Drosophila* express different odorant receptors, which determine the range of odorants that the neurons sense. Axons from each class of olfactory neurons project to a singular glomerulus in the antennal lobe, the site where olfactory information is first processed in the brain (Gomez-Diaz et al., 2018).

Environmental odors contain an enormous variety of molecular structures, underlying the need for numerous receptors to encode these stimuli. Insect genomes contain two sizeable families of insect odorant receptors, the Odorant Receptors (ORs) and Ionotropic Receptors (IRs) (Robertson, 2019; Supplementary Figure 1). Most IRs respond best to carboxylic acids and amines, whereas the ORs respond to esters, alcohols, ketones, and aromatics as well as long-chain hydrocarbon pheromones (Gomez-Diaz et al., 2018; Yan et al., 2020; Ni, 2021). Some members of the IR family alternatively function as taste, humidity, or temperature receptors (Ni, 2021). Both ORs and IRs form tetrameric odorant-gated cation channels (Sato et al., 2008; Smart et al., 2008; Abuin et al., 2011; Butterwick et al., 2018). Each OR subunit has seven transmembrane domains, with a hydrophobic ligand-binding pocket located in the transmembrane region (Butterwick et al., 2018; Del Marmol et al., 2021). The IRs are distantly related to the glutamate receptor family, with each subunit containing an extracellular ligand-binding domain, three transmembrane domains, and a re-entrant pore loop (Abuin et al., 2011, 2019).

The ORs form heteromeric ion channels comprised of an obligate subunit, Odorant Receptor Co-Receptor (Orco), and one of many potential odorant-binding tuning subunits, “OrX”, that define the odors to which the receptor can respond (Larsson et al., 2004; Neuhaus et al., 2005; Benton et al., 2006; Hallem et al., 2006; Sato et al., 2008). OrX subunits produce only minimal odor responses when expressed in heterologous cells in the absence of Orco, and odor sensitivity of OrX-expressing olfactory neurons is abolished in *Orco* mutants (Larsson et al., 2004; Neuhaus et al., 2005; Sato et al., 2008; Smart et al., 2008). In the absence of Orco *in vivo*, OrX protein is unstable and mostly unable to traffic to the ciliated neuronal dendrites (Larsson et al., 2004; Benton et al., 2006). The Orco co-receptor is highly conserved across insect species, whereas the tuning receptors are highly variable and evolve to detect odorants in different ecological niches (Jones et al., 2005; Robertson, 2019).

Similarly, IR receptors are heteromers comprised of one of many odorant-binding tuning subunits, “IrX”, and one or more co-receptors, Ir8a, Ir25a, and Ir76b (Abuin et al., 2011; Ni, 2021; Vulpe and Menuz, 2021). As with the OR family, the formation of functional IR receptors requires co-receptors in heterologous cells and *in vivo* (Abuin et al., 2011, 2019). Studies in *Drosophila* indicate that neuronal responses to carboxylic acids are lost in *Ir8a* mutants, whereas amine responses are lost in either *Ir25a* or *Ir76b* mutants (Abuin et al., 2011; Vulpe and Menuz, 2021). Ir8a and Ir25a contain an amino-terminal domain (ATD) predicted to affect receptor assembly, and an extracellular “co-receptor extra loop” (CREL) domain involved in ciliary trafficking of receptors (Croset et al., 2010; Abuin et al., 2011, 2019). Ir25a and Ir8a co-receptors are well conserved across insects (Croset et al., 2010; Rytz et al., 2013). In contrast, the Ir76b co-receptor does not contain the ATD or CREL domains and is less well-conserved (Croset et al., 2010).

Recent data suggest that the impact of Orco goes beyond odorant receptor protein function and localization to also play a neurodevelopmental role. *Orco* mutation in ants, honeybees, and *Manduca* moths induces a decrease in the volume and number of antennal lobe glomeruli (Trible et al., 2017; Yan et al., 2017; Fandino et al., 2019; Chen et al., 2021). This neurodevelopmental defect is associated with a loss of olfactory neurons in the antenna in ants and reduced antennal expression of most OR tuning receptors in honeybees (Trible et al., 2017; Yan et al., 2017; Chen et al., 2021). In contrast, mutation of *Orco* does not affect the gross anatomy or volume of the antennal lobe in *Drosophila*, the species with the best characterized olfactory system (Larsson et al., 2004; Chiang et al., 2009; Trible et al., 2017). There are only limited data on whether *Orco* impacts olfactory neuron survival in the *Drosophila* antenna. Olfactory neurons were reported to have generally normal morphology, and loss of olfactory neurons may be less prevalent than in other species (Larsson et al., 2004). For example, the number of antennal Or47b-expressing olfactory neurons is only ∼25% reduced at 14 days post-eclosion (DPE), and there is no loss of Or22a-expressing neurons at 6 DPE (Chiang et al., 2009; Hueston et al., 2016).

However, one study reported a widespread and progressive retraction of olfactory neuron axons in *Drosophila Orco* mutants, consistent with the initial stages of neuronal degeneration (Chiang et al., 2009). Further, a recent study found that olfactory neurons in the *Drosophila* maxillary palp progressively degenerate in *Orco* mutants, with more than 60% lost by 15 days post-eclosion (Task and Potter, 2021). This degradation differentially affects palp olfactory neuron classes, with nearly complete loss of Or46a-expressing neurons but no loss of Or42b-expressing neurons. Similarly, the expression of some OR tuning receptors in the antenna is decreased at 4 DPE in the absence of Orco (Jafari et al., 2021). Considering that olfactory neuron loss may be progressive with age and/or class-dependent, antennal olfactory neuron degeneration may have been overlooked previously.

Here, we set out to systematically and comprehensively examine the role of odorant co-receptors on the survival of antennal olfactory neurons in *Drosophila*. We use antennal RNASeq to identify OR and IR tuning subunits whose expression is lost in co-receptor mutants, as an indicator of olfactory neuron classes that may have degenerated. We then used transgenic reporters and lineage tracing to determine the fate of multiple specific olfactory neuron classes. Together, our data reveal that loss of odorant co-receptors leads to a decrease in mRNA expression of many tuning receptors, but few antennal olfactory neurons degenerate as a consequence of this insult.

## Materials and methods

### Fly stocks

Co-receptor mutant lines were obtained from the Bloomington Drosophila Stock Center: #23130 *Orco^2^*, #41744 *Ir8a^1^*, #51309 *Ir76b^1^*, and #41737 *Ir25a^2^*. Each of these mutations was outcrossed for at least ten generations into *wCS*, our standard genetic background that is a *Cantonized w^1118^* line (Koh et al., 2014). Such flies also served as the control flies (WT) for RNASeq experiments. We also obtained driver and reporter lines from the Bloomington Drosophila Stock Center: #9946 *Or13a-GAL4*, #9947 *Or19a-GAL4*, #24614 *Or49b-GAL4*, #23125 *Or82a-GAL4*, #23131 *Or83c-GAL4*, #41732 *Ir64a-GAL4*, #32219 *10XUAS-IVS-mCD8:RFP*, and #28280 *UAS-G-TRACE* (*UAS-RedStinger,UAS-FLP.D,UBI-p63E(FRT.STOP)Stinger*). The *Ir75a-GAL4* line was reported previously (Vulpe et al., 2021). Specific genotypes of fly lines used in this study are provided in Supplementary Table 1.

### RNA isolation and sequencing

**FIGURE 1:**
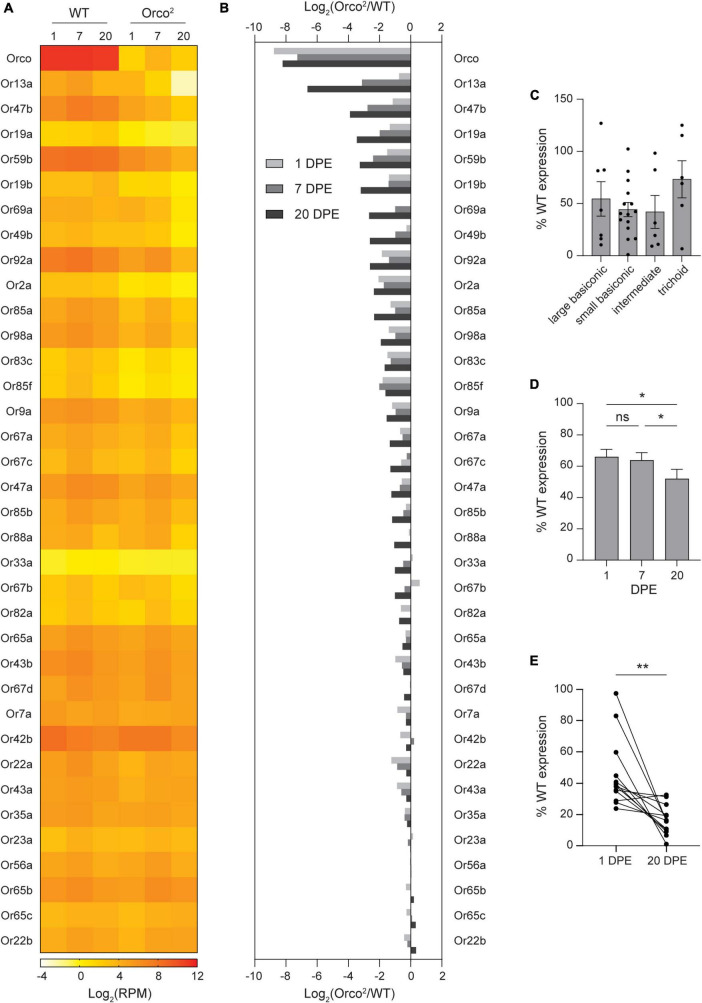
Expression of select OR tuning receptors is progressively lost in *Orco^2^* flies. **(A)** Heat map showing the average antennal expression in Log_2_(RPM) for each of the known antennal OR genes at 1, 7, and 20 DPE in WT and *Orco^2^* flies. **(B)** Bar graph reporting the Log_2_ of the expression ratio between *Orco^2^* and WT flies for members of the OR family. ORs are arranged in descending order of the Log_2_ expression ratio at 20 DPE. **(C)** Expression of antennal OR tuning receptors in *Orco^2^* flies as a percentage of expression in WT flies at 20 DPE. **(D)** Average expression of antennal OR tuning receptors in *Orco^2^* flies as a percentage of expression in WT flies at different ages. **(E)** Graph showing expression of each of the 13 strongly reduced OR tuning receptors at 1 and 20 DPE in *Orco^2^* flies as a percentage of expression in WT flies. Statistical significance is presented as *p < 0.05 and **p < 0.01. Other comparisons were not significant (*p* > 0.05).

Antennae were dissected from flies either 0–2 DPE (“1 DPE”), 4–9 DPE (“7 DPE”), or 16–20 DPE (“20 DPE”), with approximately equal numbers of males and females. Samples were collected from wCS (WT), *Ir25a^2^*, *Ir8a^1^*, *Ir76b^1^*, *Orco^2^* and *Ir25a^2^;Ir76b^1^* flies at the time points specified in the text. WT samples of the same age were compared to *Orco^2^* flies in Figure 1 and to IR co-receptor mutants in Figure 5. We isolated RNA using a protocol described previously (Mohapatra and Menuz, 2019). In brief, flies were dipped in liquid nitrogen, and antennae were manually dissected into 1.5 mL Eppendorf tubes in a liquid nitrogen bath. Antennae from 150 to 200 flies were dissected for each sample. Antennae were homogenized using disposable RNAse free micropestles (USA Scientific) and QIAshredder columns (Qiagen). RNA was extracted using an RNAeasy Micro Kit (Qiagen) and digested with DNAse from an iScript gDNA Clear cDNA Synthesis Kit (Bio-Rad). The Center for Genome Innovation at the University of Connecticut carried out quality control on the RNA samples (∼0.5 μg each) and generated libraries with the TruSeq Stranded mRNA library kit (Illumina). Sequencing produced ∼10.3–30.3 million paired-end reads per sample.

### RNASeq quantification and differential expression analysis

Raw reads were stored in BaseSpace Sequence Hub (Illumina) and downloaded to the University of Connecticut High Performance Computing cluster. Trimmed reads were generated using Sickle version 1.33 with the default parameters (Joshi and Fass, 2011). The *Drosophila* gene annotation file and reference genome Release 6.46 were downloaded from NCBI (dos Santos et al., 2014; Gramates et al., 2022). Trimmed reads were aligned to the reference genome with mitochondrial genes removed using STAR with the default parameters (Dobin et al., 2013). Mapped reads generated by STAR are available under BioProject accession number PRJNA735732 at the NCBI Sequence Read Archive (SRA). Raw counts of reads mapping uniquely to each gene were generated using HTSeq (Anders et al., 2015). HTSeq-count was used with “intersection-non-empty” and “non-unique-none” modes to handle reads mapping to more than one feature. To analyze expression of Ir76b on an exon-by-exon basis, we fed the aligned reads from STAR into DEXSeq and ran the program with the default parameters to estimate the RPM for each exon (Anders et al., 2012; Reyes et al., 2013). Three independent biological replicates were analyzed for most genotypes and ages. Because samples were not included for analysis if they were contaminated with other body parts (ex. heads), we utilized only two *Ir76b^1^* 7 DPE and *Orco^2^* 20 DPE samples. Further analysis was carried out on 1, 7, and 20 DPE samples using the raw gene counts from HTSeq-count with the EdgeR v3.40.2 package in R studio v4.2.1 (Robinson et al., 2010). First, the Reads per Million Mapped Reads (RPM) value for each gene in each sample was calculated using EdgeR (Dataset S1, “RPM”). These values were used for all figures, except in Figure 5A. In that figure, a small value (0.05) was added to each RPM value when calculating the Log_2_(RPM) values to avoid undefined values resulting from zero expression of *Ir8a* in *Ir8a^1^* mutants.

For differential gene expression analysis on 7 DPE samples, a general linearized model in EdgeR was first used to identify differentially expressed (DE) genes over all samples (Dataset S1, “GLM”). To identify the specific samples in which the genes were differentially expressed, an EdgeR ExactTest was run between each co-receptor mutant and WT in a pair-wise fashion (Dataset S1, “ExactTest Orco vs WT”, “ExactTest Ir8a vs WT”, “ExactTest Ir25a vs WT”, “ExactTest Ir76b vs WT”, and “ExactTest Ir25a;Ir76b vs WT”). We identified DE genes for each co-receptor mutant as those with FDR < 0.01 in both the GLM and ExactTest and with a > 2-fold change in expression (Dataset S1, “all DE genes”). Finally, we identified 206 protein-coding DE genes that were differentially expressed in at least one genotype (Dataset S1, “206 protein encoding DE genes”).

DAVID with Knowledgebase v2023q2 was used for functional annotation of the 206 DE genes (Dataset S1, “206 protein encoding DE genes”) (Sherman et al., 2022). We used DAVID Functional Annotation Clustering to determine the types of functions that are statistically enriched among the DE genes in each co-receptor mutant relative to WT, with separate analyses run for upregulated and downregulated DE genes. We considered terms significant if they had a Benjamini and Hochberg adjusted p-value of <0.01. We used FlyBase (Release 6.54) to update the FlyBase Gene ID numbers and Gene Symbols for each gene in Dataset S1 “206 protein encoding DE genes” (Gramates et al., 2022).

### Antennal whole-mount imaging

Fly crosses were used to generate experimental flies with either the WT or co-receptor background. For OrX > RFP and OrX > G-TRACE experiments, antennae were dissected from male and female flies either 1–2 DPE (“1 DPE”) or 17–22 DPE (“20 DPE”). For Ir75a > RFP and Ir64a > G-TRACE, antennae were dissected from 7 to 9 DPE (“7 DPE”) male flies. Dissected antennae were washed in 1X PBS, and then mounted in VectaShield (Vector Labs). Whole-mounted antennae were imaged on a Nikon A1R confocal microscope in the University of Connecticut Advanced Light Microscopy Facility. RFP-labeled antennae were imaged within 4 h after dissection, and G-TRACE antennae were imaged within 45 min after dissection, due to the weak expression of nuclear EGFP. Stacks of images (0.5 μm z-step) were acquired with a 40x oil-immersion objective. The imaging gain and offset were set the same for each compared pair of fly lines (same driver, reporter, and age, but either mutant or WT background). Images were analyzed with ImageJ/Fiji software using the Cell Counter tool (Schindelin et al., 2012).

### Antennal immunohistochemistry

Immunohistochemistry was carried out on male and female flies. Flies were 17–21 DPE for anti-elav, anti-cleaved-caspase-3 and anti-cleaved-Dcp-1 staining and 6-8 DPE for anti-Ir64a staining. Approximately 4–5 flies of each sex were anesthetized, placed in an alignment collar, encased with OCT (Tissue-Tek) in a silicone mold, and frozen over dry ice. Blocks containing ∼8–10 aligned heads were snapped off and stored in 1.5 mL microcentrifuge tubes at −80°C until sectioning. A Leica CM3050S cryostat was used to collect 20 μm thick tissue sections at −20°C. All further steps were at room temperature, unless noted. Tissue sections were fixed for 10 min in a solution of PBS containing 4% paraformaldehyde. Sections were washed 3 × 5 min with PBS, permeabilized with PBS containing 0.2% TritonX-100 (PBST) for 30 min and blocked with PBST containing 1% Bovine Serum Albumin (BSA) for 30 min. The primary antibodies were diluted into 1% BSA-PBST, and 200 μL of the antibody solution was applied to each slide under a bridged coverslip. Primary antibodies were rabbit anti-cleaved-caspase-3 (1:500, RRID:AB_2341188), rabbit anti-cleaved-Dcp-1 (1:500, RRID:AB_2721060), rat anti-elav (1:10, RRID:AB_528218), and rabbit anti-Ir64a antibody (1:100, RRID:AB_2566854). Tissue sections were incubated overnight at 4°C, before being washed 3 × 10 min in PBST. Then, sections were incubated for 2 h in the dark with 200 μL of the secondary antibody solution composed of the appropriate secondary antibody diluted 1:500 into 1% BSA-PBST. Secondary antibodies were goat anti-rabbit Alexa Fluor 568 (RRID:AB_143157) for the caspase antibodies, goat anti-rat Alexa Fluor 488 (RRID:AB_2534074) for anti-elav, and goat anti-rabbit Alexa Fluor Plus 555 (RRID:AB_2633281) for anti-Ir64a. Slides were then washed 3 × 5 min, air dried for 30 min in the dark, and mounted in VectaShield (Vector Labs).

Stained antennal sections were imaged on a Nikon A1R confocal microscope. Stacks of images (0.5 μm z-step) were acquired with a 40x oil-immersion objective. ImageJ/Fiji software was used to analyze antennal histology. For anti-cleaved-caspase-3, anti-cleaved-Dcp-1, and anti-elav staining, an eleven-slice volume from each antenna was selected in the middle of the stack. All labeled cells in the volume were counted for anti-cleaved-caspase-3 and anti-cleaved-Dcp-1 quantification. Due to the density of elav staining, the number of elav^+^ cells in the eleven-slice volume was quantified in the region under a ∼1100 μm^2^ rectangle placed over the distal region of the antenna. For quantification of Ir64a^+^ neurons, only antennal sections in which the third chamber of the sacculus was visible with DIC were used because this region contains Ir64a^+^ neurons (Ai et al., 2010). Here, 20 slice volumes centered on the sacculus were used for quantification, and all cells in the volume were counted.

### Statistical analysis

Data were analyzed using GraphPad Prism 10. Bar graphs depict the mean ± SEM overlaid with the individual data points. Student’s *t*-tests or a paired t-test were used to compare two conditions as described in the text. A one-way ANOVA or repeated measures ANOVA followed by Tukey’s *post hoc* test was used for comparisons of multiple genotypes, as noted in the text. Differences were considered significant if *p* < 0.05. Values are given as the mean ± SEM.

## Results

### Expression of select tuning OrXs is progressively reduced in *Orco^2^* mutants

We reasoned that any classes of olfactory neurons that degenerate in the absence of Orco can be revealed by a loss of mRNA expression for their associated OR tuning receptors. Transcriptomic profiling can be used to quantitatively compare tuning receptor expression at multiple time points on all antennal olfactory neuron classes in parallel. We therefore used RNA Sequencing to compare antennal expression of OR tuning receptors in wild-type and *Orco^2^* mutants, in which *Orco* expression is abolished. The *Orco^2^* line was backcrossed for ten generations to our control white-eyed Canton-S flies (WT) to minimize genetic variability between lines. Antennal RNA harvested from the WT and *Orco^2^* flies at 1, 7, and 20 DPE was subjected to next-generation transcriptional profiling, and receptor expression was quantified with HTSeq (Dataset S1, “RPM”). As expected, *Orco* expression is nearly eliminated in *Orco^2^* flies (< 1% of WT at all ages) (Figures 1A, B). Remaining Orco expression is most likely derived from the exons that remain in the *Orco^2^* flies because the mutation replaces only the first five transmembrane domains with a mini-white marker, and a smaller downstream portion of the gene remains in the genome.

We examined the expression of the 35 known antennal OR tuning receptors that are expressed in the antenna. We found that the loss of *Orco* leads to reduced OR tuning receptor expression (Figures 1A, B), with average OrX expression 52% of WT levels at 20 DPE. In contrast, expression of IR tuning receptors is maintained in *Orco^2^* antenna, with average IrX expression 104% of WT levels at 20 DPE (Supplementary Figure 2).

The average loss of OR tuning receptors obscures a wide range of individual receptor variability. The greatest reduction is seen for Or13a, whose expression in *Orco^2^* mutants is 1% of WT at 20 DPE (Figures 1A, B). Other tuning receptors with less than 1/3rd of WT level expression are *Or47b* (7%), *Or19a* (9%), *Or59b* (10%), *Or19b* (11%), *Or69a* (16%), *Or49b* (16%), *Or92a* (16%), *Or2a* (19%), *Or85a* (20%), *Or98a* (26%), *Or83c* (31%), and *Or85f* (33%). On the other extreme, expression of 10 tuning receptors remains at >80% of WT level at 20 DPE: *Or7a*, *Or42b*, *Or22a*, *Or43a*, *Or35a*, *Or23a*, *Or56a*, *Or65b*, *Or65c*, and *Or22b*. The identity of the tuning receptors whose expression is strongly Orco-dependent is not explained by sensillar morphology. The 13 tuning receptors with strongly reduced expression are found in all morphological classes of Orco-expressing sensilla: large and small basiconic, intermediate, and trichoid sensilla. Likewise, there is no difference in average tuning receptor loss by sensilla morphology (one-way ANOVA *p* > 0.05) (Figure 1C).

Loss of OR tuning receptor expression is generally progressive. On average, OR tuning receptor expression in *Orco^2^* mutants relative to that in WT flies declines mildly with age (repeated measures one-way ANOVA followed by Tukey’s *post hoc* test *p* < 0.05, *n* = 35 receptors) (Figure 1D). The progressive nature of tuning receptor loss is clearer when considering only those receptors with strongly reduced expression at 20 DPE. For example, 60% of Or13a expression remained at 1 DPE, 11% at 7 DPE, and only 1% at 20 DPE (Figures 1A, B). The average expression in *Orco^2^* mutants of the 13 most strongly reduced tuning receptors declined from 46% of WT at 1 DPE to 17% of WT at 20 DPE (paired t-test, *p* < 0.01, *n* = 13 receptors) (Figure 1E).

### Reduced OrX expression is rarely associated with olfactory neuron degeneration

**FIGURE 2:**
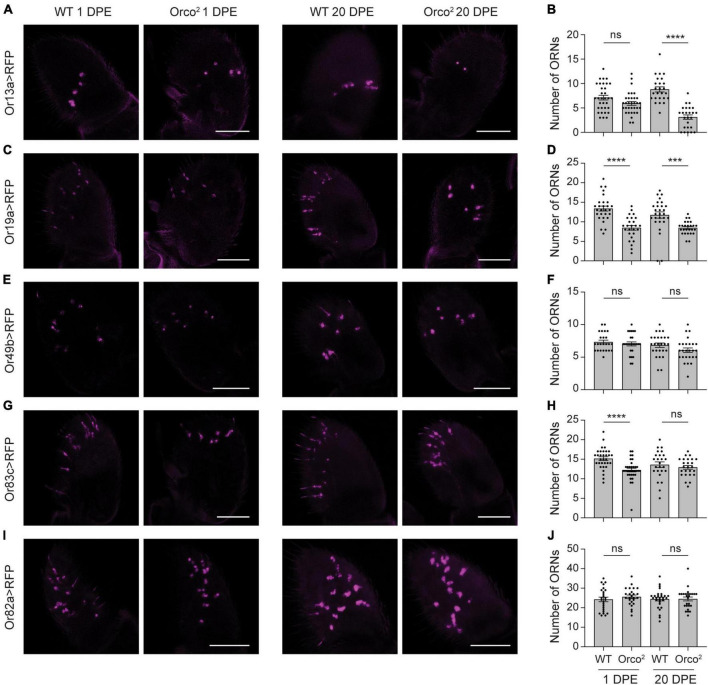
Reductions in OrX expression are sometimes associated with mild loss of associated olfactory neurons. **(A)** Whole-mount images of antenna taken from 1 and 20 DPE flies in which UAS-mCD8:RFP is driven by Or13a-GAL4 in a WT or *Orco^2^* mutant background. Scale bar, 50 μm. **(B)** Quantification of the number of olfactory receptor neurons (ORNs) detected in each antenna. Bar graph depicts the mean ± SEM overlaid with the individual data points. **(C–J)** Similar but using the Or19a-GAL4 **(C,D)**, Or49b-GAL4 **(E,F)**, Or83c-GAL4 **(G,H)**, and Or82a-GAL4 **(I,J)** drivers. Statistical significance is presented as ***p < 0.001, ****p < 0.0001, and ns *p* > 0.05.

We wondered whether the loss of OrX expression was due to the degeneration of antennal olfactory neurons in *Orco^2^* mutants, similar to what was previously observed in the maxillary palp in *Drosophila* and the antenna in ants (Trible et al., 2017; Task and Potter, 2021). To investigate the effects of Orco on the survival of specific classes of antennal olfactory neurons, we drove *UAS-mCD8:RFP* expression using validated *OrX-GAL4* drivers in WT flies and *Orco^2^* mutants and quantified the number of labeled olfactory neurons in whole mount antennae. We first examined neurons that express Or13a, the tuning receptor with the greatest reduction in expression in *Orco^2^* mutants at 20 DPE. Examination of Or13a > RFP antennae revealed a ∼65% reduction in the number of Or13a^+^ olfactory neurons in *Orco^2^* flies at 20 DPE (Student’s t-test *p* < 0.0001, *n* = 25 antenna each) (Figures 2A, B). The loss of Or13a^+^ neurons is progressive, as there is no significant difference in the number of neurons at 1 DPE (Student’s *t*-test *p* > 0.05, *n* = 25 antenna each).

We also examined neurons that express Or19a, the receptor with the third greatest reduction in expression in *Orco^2^* flies. There is a ∼30% loss of Or19a^+^ olfactory neurons at both 1 and 20 DPE (Student’s *t*-tests *p* < 0.0001 at 1 DPE and *p* < 0.001 at 20 DPE, *n* = 26–28 antenna each) (Figures 2C, D). We next looked at Or49b^+^ and Or83c^+^ neurons, as expression of both receptors is strongly reduced in *Orco^2^* flies. We found that the number of Or49b^+^ neurons is unchanged in the absence of *Orco* (Student’s *t*-tests *p* > 0.05 at 1 and 20 DPE, *n* = 24–26 antenna each) (Figures 2E, F). Although we detected slightly fewer (∼20%) Or83c^+^ olfactory neurons at 1 DPE in *Orco^2^* flies compared to controls, there is no difference in number at 20 DPE (Student’s t-tests *p* < 0.0001 at 1 DPE and *p* > 0.05 at 20 DPE, *n* = 25–34 antenna each) (Figures 2G, H). Finally, we examined Or82a^+^ olfactory neurons as a representative for receptors with a more moderate loss of receptor expression (*Or82a*: 40% decreased versus WT at 20 DPE). Here, we found no significant difference in the number of olfactory neurons in WT and *Orco^2^* flies, both at 1 and 20 DPE (Student’s *t*-tests *p* > 0.05 at 1 and 20 DPE, *n* = 25–28 antenna each) (Figures 2I, J). There appears to be a non-linear relationship between reductions in tuning receptor expression and loss of olfactory neurons in *Orco^2^* mutants at 20 DPE (Supplementary Figure 3), with substantial (> 25%) reductions in olfactory neuron number only observed with >90% reductions in tuning receptor expression.

In these experiments, we observed that labeling of some olfactory neurons is weaker in *Orco^2^* mutants compared to WT flies (Figure 2), even if the number of cells did not change. This could be a consequence of using *OrX-GAL4* lines to label different classes of olfactory neurons. If OR tuning receptor mRNA expression is reduced, then *OrX-GAL4* expression may also be decreased. Likewise, RFP expression tended to be somewhat stronger at 20 DPE than 1 DPE, consistent with the known increase in odorant receptor expression to adult levels during the first few days post-eclosion (Jafari et al., 2021). Together, this may explain how there can be fewer Or83c^+^ cells in *Orco^2^* mutants at 1 DPE, but no change at 20 DPE. It also suggests that the loss of Or13a^+^ and Or19a^+^ olfactory neurons in *Orco^2^* mutants at 20 DPE may be overestimated.

To attempt to overcome this issue, we utilized the G-TRACE lineage labeling technique (Evans et al., 2009). In this approach, real time expression of GAL4 labels cells with nuclear marker RedStinger, and cells that have ever expressed the GAL4 are labeled with nuclear EGFP using a FLP recombinase-FRT approach. By using G-TRACE, we hypothesized that we could detect ORNs at 20 DPE, as long as they transiently express the OrX-GAL4 at a sufficient level earlier in development. The benefit of this system is that FLP recombination only needs to occur once to allow for permanent GFP-marking of ORNs, even if OrX-GAL4 expression (and therefore RedStinger expression) is very weak at the 20 DPE time point.

**FIGURE 3:**
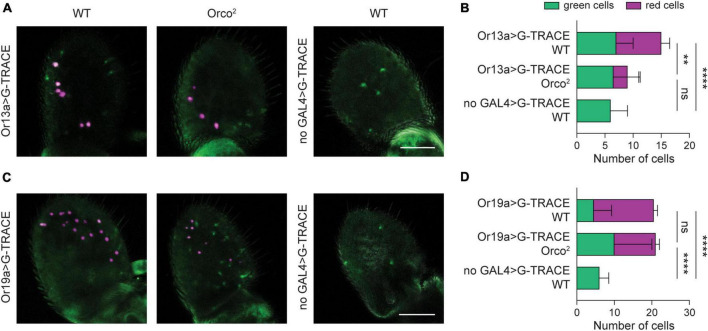
Retention of Or19a^+^ olfactory neurons despite severe loss of Or19a mRNA expression. **(A)** Whole-mount images of antenna taken from 20 DPE flies in which the G-TRACE cell lineage tracing system transgenes are driven by Or13a-GAL4 in a WT or *Orco^2^* mutant background. The right panel shows a control WT antenna in which the GAL4 is absent. Scale bar, 50 μm. **(B)** Quantification of the average number of GFP^+^ RFP^–^ cells (green cells) and RFP^+^ cells (red cells) per antenna in each genotype. Bar graph depicts the mean ± SEM. **(C,D)** Similar but using the Or19-GAL4 driver. Statistical significance for comparison of the total number of cells detected is presented as **p < 0.01, ****p < 0.0001, and ns *p* > 0.05.

We first used Or13a-GAL4 to drive the G-TRACE system. We found a ∼60% reduction in the number of red Or13a^+^ cells in *Orco^2^* mutants compared to WT flies at 20 DPE (Student’s t-test *p* < 0.0001, *n* = 17–18 antenna each) (Figures 3A, B). No RFP^+^ cells were seen in the absence of the GAL4 driver, but these control flies do have a small number of GFP^+^ cells, suggesting low-level FLP recombination in the absence of a GAL4 driver. The number of GFP^+^ RFP^–^ cells is similar in all conditions (one-way ANOVA *p* > 0.05, *n* = 17–18 antenna each) (Figures 3A, B). Accordingly, there is a significant reduction in the total number of Or13a^+^ cells in *Orco^2^* antennae compared to WT flies (one-way ANOVA followed by Tukey’s *post hoc* test *p* < 0.01, *n* = 17–18 antenna each). Together, our G-TRACE data are consistent with the partial loss of Or13a^+^ ORNs observed with the simpler RFP reporter experiments (Figures 2A, B).

We next used *Or19a-GAL4* to drive the G-TRACE system (Figures 3C, D). We found a ∼36% decrease in the number of RFP^+^ cells in *Orco^2^* flies compared to WT controls (Student’s *t*-test *p* < 0.0001, *n* = 18–19 antenna each), consistent with the loss observed earlier (Figure 2D). However, this loss is concomitant with an increase in the number of GFP^+^ RFP^–^ cells (one-way ANOVA followed by Tukey’s *post hoc* test *p* < 0.01 vs WT and *p* < 0.05 vs no-GAL4 control, *n* = 17–19 antenna each). As a result, the total number of labeled Or19a^+^ cells is unchanged in *Orco^2^* mutants (one-way ANOVA followed by Tukey’s *post hoc* test *p* > 0.05, *n* = 17–19 antenna each). Together, this suggests that reduced Or19a-GAL4 expression in *Orco^2^* mutants causes some Or19a neurons to appear RFP^–^, and that Or19a^+^ olfactory neurons persist in *Orco^2^* mutants despite > 90% loss of *Or19a* mRNA expression.

### Loss of Orco does not cause widespread olfactory neuron degeneration

**FIGURE 4:**
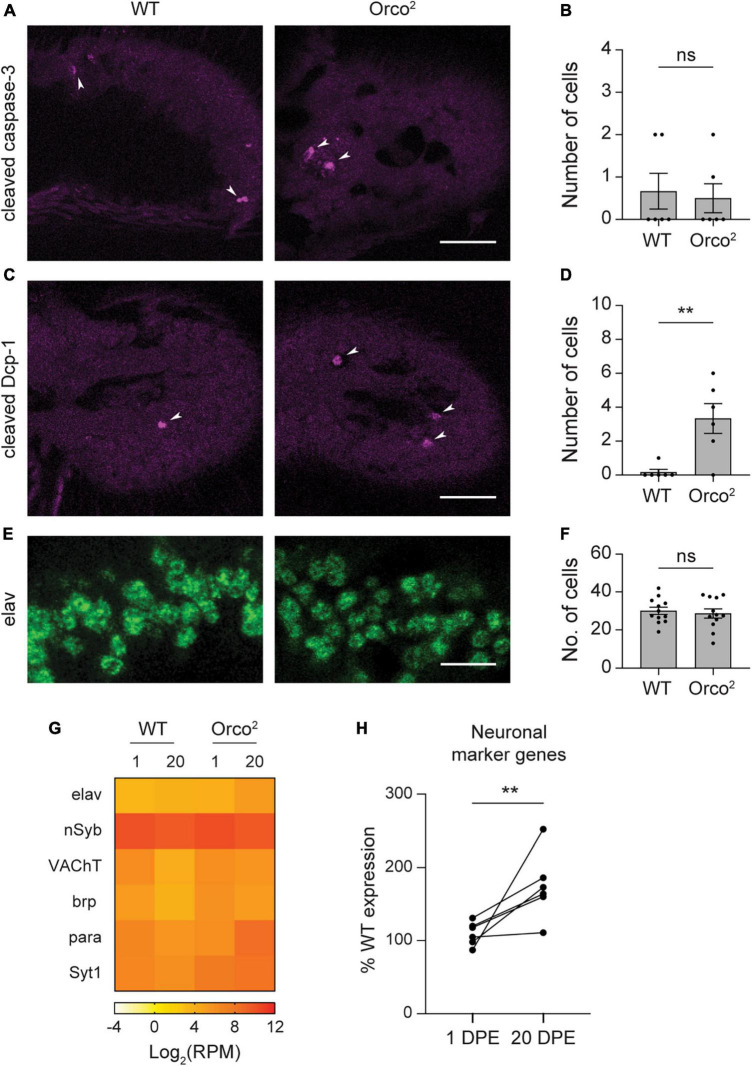
Loss of Orco does not cause widespread olfactory neuron degeneration. **(A)** Antennal sections from WT or *Orco^2^* flies stained with anti-cleaved-caspase-3. White arrowheads point to labeled cells. Scale bar, 25 μm. **(B)** Quantification of the number of labeled cells detected in each antenna. Bar graph depicts the mean ± SEM overlaid with the individual data points. **(C,D)** Similar for staining with anti-cleaved-Dcp-1. **(E,F)** Similar for staining with anti-elav. The image covers the same areas as the rectangle used for quantification through an eleven-slice volume. Scale bar, 10 μm. **(G)** Heat map showing the average antennal expression in Log_2_(RPM) for neuronal marker genes at 1 and 20 DPE in WT and *Orco^2^* flies. **(H)** Graph showing expression of each of the neuronal marker genes at 1 and 20 DPE in *Orco^2^* flies as a percentage of expression in WT flies. Statistical significance is presented as **p < 0.01 and ns *p* > 0.05.

Our data using markers for individual olfactory neuron classes suggest that most antennal olfactory neurons do not degenerate in the absence of Orco, unlike those in the maxillary palp. However, we only examined a subset of the many olfactory neuron classes. To more comprehensively investigate whether cell death occurs in *Orco^2^* flies, we used histology to look for signs of apoptosis. In flies, cleaved effector caspases serve as cell death markers (Vasudevan and Ryoo, 2016). There are four effector caspases in *Drosophila*, of which two (*Drice*/*caspase-3* and *Dcp-1*) are expressed at >1 RPM in *Orco^2^* flies at 20 DPE in the antenna; their expression level was similar to that in WT flies (Dataset S1, “RPM”). To determine if the caspase proteins were activated by cleavage, we stained antennal sections with anti-cleaved-caspase antibodies. On average, we found less than one cell labeled with anti-cleaved-caspase-3 in antenna from either WT or *Orco^2^* flies (Student’s *t*-test *p* > 0.05, *n* = 6 antenna each) (Figures 4A, B). Similarly, we detected few cells labeled with anti-cleaved-Dcp-1 in WT and *Orco^2^* antennae, although there was an increase in the absence of Orco (WT: 0.2 ± 0.2 and *Orco^2^*: 3.3 ± 0.9 cells, Student’s *t*-test *p* < 0.01, *n* = 6 antenna each) (Figures 4C, D).

To more directly determine if there is a loss of neurons in the *Orco^2^* mutants, we also compared the number of antennal cells labeled with the neuronal marker elav (Figures 4E, F). To do this, we stained antennal sections with anti-elav and compared the number of cells within a fixed volume. We found a similar number of elav-positive cells in WT and *Orco^2^* antennae (WT: 30.0 ± 1.9 and *Orco^2^*: 28.6 ± 2.4 cells, Student’s *t*-test *p* > 0.05, *n* = 12 antenna each). Consistent with this finding, expression of *elav* transcripts was not reduced in *Orco^2^* flies in our RNASeq data. Interestingly, although *elav* and other neuronal markers *nSyb*, *VACht*, *brp*, *para*, and *Syt1* generally had unchanged expression in *Orco^2^* mutants at 1 DPE, their expression was often higher at 20 DPE (paired *t*-test, *p* < 0.05) (Figures 4G, H). Together, these data suggest that the loss of OR tuning receptor expression in *Orco^2^* mutants is independent of neuron degeneration, which does not broadly occur in *Orco^2^* flies.

### Loss of IR co-receptors affects expression of associated IR tuning receptors

Does expression of IR tuning receptor mRNA also rely on expression of their associated co-receptors? Previous electrophysiology experiments have shown that amine-sensitive IR tuning receptor responses are selectively eliminated in *Ir76b*, *Ir25a*, and *Ir25a;Ir76b* mutants, whereas acid-sensitive IR tuning receptor responses are selectively eliminated in *Ir8a* mutants (Abuin et al., 2011; Vulpe and Menuz, 2021). We therefore examined IR tuning receptor expression using antennal RNASeq on outcrossed IR family co-receptor mutants at 7 DPE, a time point at which average OR tuning receptor expression in the absence of Orco is only ∼60% of that in control flies.

**FIGURE 5:**
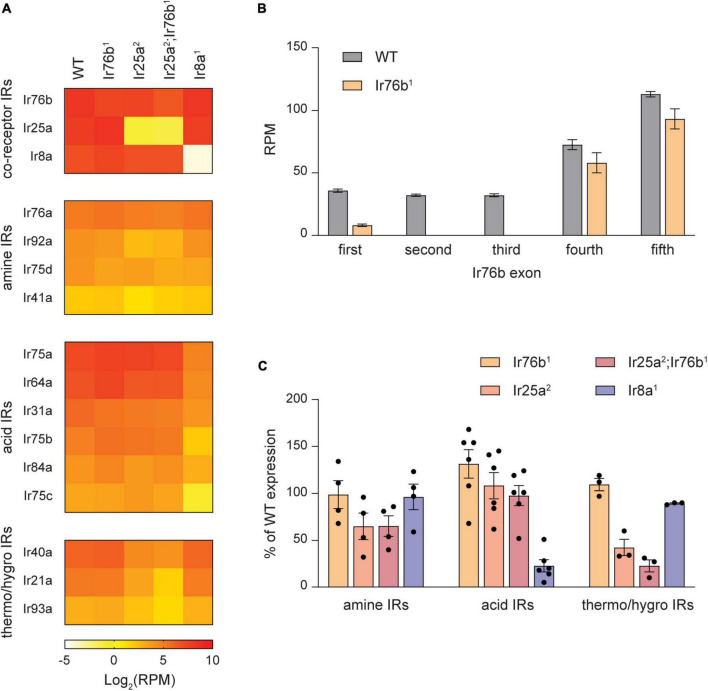
Expression of many IR tuning receptors depends on their IR co-receptor. **(A)** Heat map showing the average antennal expression in Log_2_(RPM) for detected antennal IR genes at 7 DPE in WT, *Ir76b^1^*, *Ir25a^2^*, *Ir25a^2^;Ir76b^1^*, and *Ir8a^1^* flies. The IR genes are grouped by their type: co-receptors, Ir25a- and Ir76b-dependent amine-sensitive tuning receptors, Ir8a-dependent acid-sensitive receptors, and Ir25a-dependent thermosensitive and/or hygrosensitive receptors. **(B)** The expression level in RPM for each exon of the *Ir76b* gene in WT flies and *Ir76b^1^* mutants. **(C)** Expression of amine-sensitive, acid-sensitive and thermo/hygrosensitive IR tuning receptors as a percentage of expression in WT flies in different IR co-receptor mutants.

As expected, expression of *Ir8a* and *Ir25a* is nearly eliminated in the *Ir8a^1^* and *Ir25a^2^* mutants, respectively (Figure 5A). Surprisingly, expression of *Ir76b* transcripts decreased only ∼40% in *Ir76b^1^* mutants (Figure 5A). Closer examination revealed that expression of the first three exons is mostly lacking (Figure 5B), in agreement with the loss of receptor function in this line (Vulpe and Menuz, 2021).

Interestingly, we found that expression of most amine-sensitive IR tuning receptors is reduced in *Ir25a^2^* mutants, but not *Ir76b^1^* mutants (Figures 5A, C). On average, expression of amine-sensitive IR tuning receptors in *Ir25a^2^* mutants is ∼65% of WT flies, with large variation by individual receptor. This reduction is not increased in *Ir25a^2^*;*Ir76b^1^* double mutants. Expression of amine-sensitive IrX receptors is generally unaffected by the loss of the acid co-receptor *Ir8a* (Figures 5A, C). In contrast, expression of all acid-sensitive receptors is consistently and strongly reduced in *Ir8a^1^* antennae, but unaffected by the loss of *Ir25a* or *Ir76b* (Figures 5A, C). We also noted that expression of *Ir40a*, *Ir21a*, and *Ir93a*, which form functional receptors with Ir25a in antennal hygrosensory and thermosensory neurons (Ni, 2021), depends on Ir25a expression. As expected, OR tuning receptor expression is mostly unaffected by the loss of IrX co-receptors (Supplementary Figure 4). Together, our data indicate that expression of many IR tuning receptors relies on expression of their specific IR co-receptors, like the reliance of OR tuning receptor expression on Orco.

### Ir8a-dependent acid-sensitive olfactory neurons mostly persist in *Ir8a^1^* flies

**FIGURE 6:**
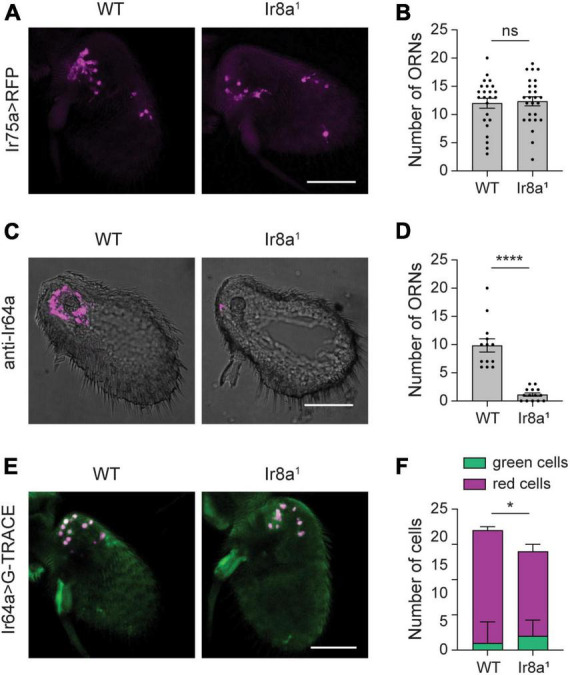
Little to no loss of Ir8a-dependent ORNs in *Ir8a^1^* flies. **(A)** Whole-mount images of antenna taken from flies in which UAS-mCD8:RFP is driven by Ir75a-GAL4 in a WT or *Ir8a^1^* mutant background. **(B)** Quantification of the number of olfactory receptor neurons (ORNs) detected in each antenna. **(C)** Antennal section from WT or *Ir8a^1^* flies stained with anti-Ir64a. **(D)** Quantification of the number of ORNs detected in each antennal section. **(E)** Whole-mount images of antenna taken flies in which the G-TRACE cell lineage tracing system transgenes are driven by Ir64a-GAL4 in a WT or *Ir8a^1^* mutant background. **(F)** Quantification of the average number of GFP^+^ RFP^–^ cells (green cells) and RFP^+^ cells (red cells) per antenna in each genotype. Scale bars, 50 μm. Bar graphs depict the mean ± SEM. Those in panels **(B,D)** are overlaid with the individual data points. Statistical significance is presented as *p < 0.05, ****p < 0.0001, and ns *p* > 0.05.

Given the pronounced reduction in Ir8a-dependent acid-sensitive tuning receptor expression in the absence of *Ir8a* (∼75%), we investigated whether this is due to the degeneration of the olfactory neurons that express these receptors. We first investigated Ir75a^+^ olfactory neurons at 7 DPE, as *Ir75a* transcript expression is only 18% of WT flies in *Ir8a^1^* mutants at this age. Using *Ir75a-GAL4* to drive *UAS-mCD8:RFP* we found no change in the number of neurons in *Ir8a^1^* antenna (Student’s *t*-test *p* > 0.05, *n* = 24–26 antenna each) (Figures 6A, B).

We next examined Ir64a, a tuning receptor with 80% loss of transcript expression in *Ir8a^1^* mutants. Here, we took advantage of a validated anti-Ir64a antibody to use immunohistochemistry to quantify the number of Ir64a-expressing neurons. We found that the number of olfactory neurons that express Ir64a protein is sharply lower (89%) in *Ir8a^1^* mutants compared to controls (Student’s *t*-test *p* < 0.0001, *n* = 13–14 antennal sections each) (Figures 6C, D). However, there could be an apparent loss of neurons if loss of Ir64a protein expression makes it difficult to detect weakly labeled neurons. Therefore, we used *Ir64a-GAL4* to drive the G-TRACE system to mark Ir64a^+^ olfactory neurons. There is a small (24%), but significant, loss of RFP^+^ neurons in *Ir8a^1^* antenna (Student’s *t*-test *p* < 0.001, *n* = 17–18 antenna each) (Figures 6E, F). This is not accompanied by a significant increase in the number of GFP^+^ RFP^–^ cells (Student’s *t*-test *p* > 0.05, *n* = 17–18 antenna each), but rather by a ∼18% loss of total labeled cells (Student’s *t*-test *p* < 0.05, *n* = 17–18 antenna each) (Figures 6E, F). Thus, loss of IR tuning receptor mRNA and protein expression is much greater than the loss of olfactory neurons in *Ir8a^1^* mutants, like what is observed in *Orco^2^* mutants.

### Changes in gene expression associated with loss of odorant co-receptors

**FIGURE 7:**
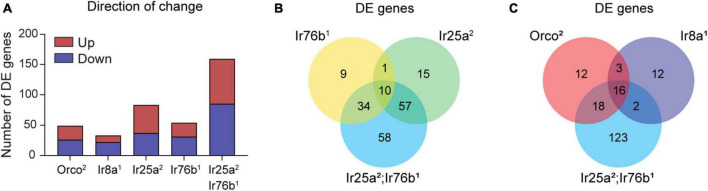
Differentially expressed genes are detected in co-receptor mutants. **(A)** Bar graph depicting the total number of DE genes identified in *Orco^2^*, *Ir8a^1^*, *Ir25a^2^*, *Ir76b^1^*, and *Ir25a^2^;Ir76b^1^* mutants. Upregulated and downregulated genes in mutant flies relative to WT are shown in maroon and navy, respectively. **(B,C)** Venn diagrams showing the overlap between DE genes detected in the different co-receptor mutant lines.

Is there a broader reorganization of gene expression in the absence of odorant co-receptors? To address this question, we used EdgeR to identify genes whose expression is differentially expressed in the antennae of co-receptor mutant flies compared to the genetic control line, focusing on those genes with at least a two-fold difference in expression. We detected a total of 206 DE protein-coding genes across all genotypes, with 33 to 159 DE genes per genotype (Figure 7A). There are roughly even numbers of upregulated and downregulated genes in each genotype. Many genes (113 of 206) are differentially expressed in at least two genotypes. For 111 of these genes, the direction of change (upregulation or downregulation) relative to control flies is the same in all genotypes in which it is differentially expressed.

We first examined the DE genes from flies lacking co-receptors for amine-sensitive IrX receptors (Figure 7B). Interestingly, the 54 DE genes in *Ir76b^1^* flies are mostly distinct from the 83 genes in *Ir25a^2^* flies, despite their similar role as co-receptors. This is aligned with our earlier finding that amine tuning receptor transcript expression depends on Ir25a, but not Ir76b (Figure 5C). It may also reflect the involvement of Ir25a, but not Ir76b, in antennal receptors mediating humidity and temperature sensing (Enjin et al., 2016; Knecht et al., 2016). Most genes (∼81%) that are differentially expressed in either *Ir25a^2^* or *Ir76b^1^* flies are also differentially expressed in the *Ir25a^2^*;*Ir76b^1^* double mutants. However, approximately one-third of DE genes in the double mutants are not differentially expressed in either *Ir25a^2^* or *Ir76b^1^* flies (Figure 7B). This may indicate that there is some level of functional redundancy between Ir25a and Ir76b in their impact on antennal olfactory neuron function and gene transcription.

We next compared the DE genes in *Orco^2^* flies and *Ir8a^1^* flies with the DE genes in *Ir25a^2^*;*Ir76b^1^* double mutants, as the latter includes most DE genes in *Ir25a^2^* or *Ir76b^1^* single mutants. We found that ∼77% of genes that are DE in *Ir25a^2^*;*Ir76b^1^* double mutants are not DE in either *Orco^2^* flies or *Ir8a^1^* flies. There is a core group of ∼16 genes that are DE in each of these genotypes relative to the control flies (Figure 7C). For 15/16 of the genes, the direction of change (upregulation or downregulation in the mutants) is consistent across all three mutant genotypes. One of these genes is *white (w)*, whose upregulation relative to the *w-* WT flies is expected because the *Orco^2^*, *Ir8a^1^* and *Ir25a^2^* mutations contain a mini-white marker that restores *w* function. The other 15 shared DE genes have disparate functions, ranging from dehydrogenase enzymes to a tRNA-synthetase to a TRP channel (Dataset S1, “206 protein encoding DE genes”). The transcription of these genes may generally be affected by the loss of odorant co-receptors or olfactory signaling. Alternatively, their differential expression may be due to the absence of *w* in the control flies, or due to slight variations in the genetic background of the control line that developed after out-crossing. Other than the 16 shared DE genes, there is little overlap between the *Ir8a^1^* DE genes and DE genes in either *Orco^2^* or *Ir25a^2^*;*Ir76b^1^* flies. However, more than half of the *Orco^2^* DE genes are also DE in the *Ir25a^2^;Ir76b^1^* double mutants.

We annotated the predicted functions of the 206 DE protein-encoding genes using DAVID (Dataset S1, “206 protein encoding DE genes”) (Sherman et al., 2022). We found that 16 are members of the OR and IR family. Other DE genes have a variety of predicted functions, including ion channels, transporters, receptors, kinases, and metabolic enzymes. We used DAVID Functional Annotation Clustering to determine what types of functions are statistically enriched among the DE genes in each genotype. There is only one significant term cluster associated with the DE genes downregulated in *Orco^2^* flies, “Drosophila Olfactory Receptor”, consisting of OR tuning receptors. Similarly, the only significant term cluster among DE genes downregulated in *Ir8a^1^* flies related to “Ionotropic Glutamate Receptor”, the IR tuning receptors. No term clusters are enriched among the upregulated DE genes in the *Ir8a^1^* or *Orco^2^* flies, nor among up- or downregulated genes in *Ir76b^1^* flies.

The upregulated genes in both *Ir25a^2^* and *Ir25a^2^*;*Ir76b^1^* flies are enriched for genes associated with “Innate Immunity”. These included several members of the Bomanin family of small, secreted peptides that can be released into the hemolymph upon microbial infection. The downregulated genes in *Ir25a^2^* flies are enriched for genes associated with the ligand “Heme”, including members of the cytochrome p450 family such as *Cyp4d21*, *Cyp4p2*, *Cyp6a17*, and *Cyp6g2*. This family is also nearly significantly enriched among the downregulated DE genes in *Ir25a^2^*;*Ir76b^1^* flies (adjusted p-value 0.084). Cyps are thought to function as Odorant Degrading Enzymes (ODEs) within the antenna, and their transcription can be upregulated by their substrates such as odorants (Baldwin et al., 2021). However, it is unclear why they are downregulated in the absence of *Ir25a*, an effect not seen in other co-receptor mutants. The term “Ion Channel” is also enriched amongst the downregulated DE genes in *Ir25a^2^* flies. In addition to members of the IR family, these include the TRP channel TrpA1 and two members of the DEG/ENaC family of sodium channels ppk14, and ppk25. However, this term is not enriched among the downregulated genes in *Ir25a^2^*;*Ir76b^1^* double-mutants. Taken together, our term enrichment analysis suggests that the major transcriptional effect seen in co-receptor mutants is on associated tuning receptors, and that large-scale transcriptional remodeling does not occur.

## Discussion

Previous work has suggested that *Drosophila* olfactory receptor neurons degenerate in *Orco* mutants due to a loss of neuronal activity, as evidenced by axonal degeneration and retraction during the first week post-eclosion (Chiang et al., 2009). Loss of olfactory neuron firing activity is thought to activate Gsk-3β kinase, a master regulator affecting neuronal morphology and synapse formation (Chiang et al., 2009; Aimino et al., 2023; Lai et al., 2023). Likewise, expression of the neuronal marker elav is strongly decreased in the maxillary palp in *Orco* mutants, suggesting widespread loss of olfactory neurons (Task and Potter, 2021). In contrast, our transcriptomic data suggest that the antennal transcriptome is not broadly remodeled in the absence of *Orco*, and that the most prominent category of differentially expressed genes are OR tuning receptors. Furthermore, there is no evidence of widespread apoptosis or loss of elav^+^ cells in *Orco^2^* flies. Thus, our data show that the absence of *Orco* does not lead to substantial loss of olfactory neurons in the antenna, at least through the first 3 weeks post-eclosion.

We also addressed whether selected classes of olfactory neurons are lost in *Orco* mutants. This possibility was suggested by the highly variable impact of *Orco^2^* mutation on expression of different OR tuning receptors in the antennal transcriptome at 20 DPE; there is >90% loss of *Or13a*, *Or47b*, and *Or19a* on one extreme, and 10 receptors with <20% reduction on the other. Our initial studies using *OrX-GAL4* lines to label neurons with an RFP reporter suggested that there are ∼65% fewer Or13a^+^ neurons and ∼30% fewer Or19a^+^ neurons in *Orco^2^* flies. This is consistent with the ∼30% loss of Or47b^+^ neurons previously reported in *Orco* mutants (Hueston et al., 2016). For each of these three tuning receptors, the loss of OrX mRNA expression is much greater than the loss of neurons would suggest. We therefore explored the possibility that there is only an apparent loss of neurons due to transient or weak *OrX-GAL4* expression, which may parallel loss of OrX mRNA expression. Using the G-TRACE lineage tracing system, we found that *Orco^2^* flies have no loss of Or19a^+^ neurons, although there is a bonafide loss of Or13^+^ neurons. Considered together with our data showing no loss of three additional olfactory neuron classes and previous work showing no loss of Or22a^+^ olfactory neurons, our data suggest that widespread loss of antennal odorant receptor expression is mostly independent of olfactory neuron degeneration in the absence of *Orco*. This conclusion could be further validated with ORN-class specific markers whose expression is independent of Orco expression; however, to our knowledge such markers have not been reported.

We found a similar dependence of IR tuning receptor mRNA expression on their associated co-receptors, Ir25a and Ir8a. This effect is particularly pronounced for the six Ir8a-dependent acid-sensitive receptors, whose average expression is decreased ∼75% in *Ir8a^1^* flies. Like Orco-dependent olfactory neurons, our data suggest that there is little degeneration of Ir8a-dependent neurons in the absence of *Ir8a*. The loss of mRNA expression of the four Ir25a-dependent amine-sensitive receptors is weaker and more variable in *Ir25a^2^* flies. Loss of Ir25a also led to moderately reduced expression of hygrosensory and thermosensory IrX receptors, which also form functional receptors with Ir25a (Enjin et al., 2016; Knecht et al., 2016). Intriguingly, no loss of amine-sensitive IrX receptors was seen in the absence of *Ir76b*, although this co-receptor is required along with Ir25a for the formation of functional amine-sensitive receptors (Vulpe and Menuz, 2021). *Ir25a* and *Ir76b* do not have a functionally redundant role in maintaining the transcriptional expression of amine-sensitive IrXs, as elimination of both co-receptors did not lead to a further reduction in their expression than the loss of Ir25a alone.

The mechanism underlying the loss of odorant tuning receptor expression in co-receptor mutants is unclear. Lowered olfactory neuron activity induces a loss of synapses in the antennal lobe and mimics the effects of *Orco* mutation on axon degeneration (Chiang et al., 2009; Aimino et al., 2023). However, this activity-dependent mechanism cannot explain the highly variable effects of *Orco* on OrX mRNA expression because that the odor-induced activity in all OrX-dependent neurons depends on Orco. Likewise, it cannot explain why maxillary palp Or46a^+^ ORNs are nearly eliminated in *Orco^2^* mutants, whereas Or42a^+^ ORNs are unaffected (Task and Potter, 2021). An activity-dependent mechanism is also inconsistent with the loss of amine-sensitive IrX receptor expression in the absence of Ir25a, but not Ir76b, given the absence of odor-induced activity in amine-sensitive neurons in the absence of either Ir25a and Ir76b (Vulpe and Menuz, 2021). Therefore, additional factors must underlie the differential susceptibility of tuning receptors to transcript loss in the absence of family-specific co-receptors. Recent work has revealed repressive transcriptional interactions between some odorant tuning receptors in insects, one of many mechanisms that support the expression of a singular tuning receptor in a particular olfactory neuron class (Jafari et al., 2021; Mika and Benton, 2021; Mika et al., 2021; Maguire et al., 2022). It will be interesting to determine if there are also transcriptional interactions between odorant tuning receptors and their co-receptors.

Our results suggest that the role of Orco in neurodevelopment is species-specific, unlike its role in neurophysiology. Whereas *Orco* mutagenesis in two species of ants leads to a massive loss of olfactory neurons in the periphery (Trible et al., 2017; Yan et al., 2017), we found that this does not occur in *Drosophila*. It was recently reported that *Orco* mutagenesis does not lead to antennal neuron loss in hoverflies, nor does it affect expression of most OR tuning receptors in *Helicoverpa* moths (Fan et al., 2022; Wu et al., 2023). Whether the widespread reduction in OR tuning receptor expression in honeybees correlates with olfactory neuron degeneration will need to be investigated (Chen et al., 2021). *Orco* mutations have been generated in a wide variety of insects, including mosquitos, locusts, moths, and others (DeGennaro et al., 2013; Koutroumpa et al., 2016; Li et al., 2016; Yang et al., 2016; Liu et al., 2017, 2023; Fandino et al., 2019; Sun et al., 2020). It will be interesting to use these organisms as tools to examine the species-specificity of Orco-dependent olfactory neuron survival.

## Data availability statement

The datasets presented in this study can be found at the NCBI Sequence Read Archive (SRA) under BioProject accession number PRJNA735732.

## Ethics statement

The manuscript presents research on animals that do not require ethical approval for their study.

## Author contributions

TL: Conceptualization, Formal analysis, Investigation, Methodology, Writing – review and editing. PM: Conceptualization, Formal analysis, Investigation, Methodology, Writing – review and editing. SB: Formal analysis, Investigation, Writing – review and editing. KM: Conceptualization, Formal analysis, Funding acquisition, Methodology, Project administration, Resources, Visualization, Writing – original draft.
