# Supplementary material for: Odorant receptor co-receptors affect expression of tuning receptors in Drosophila
Source: Front Cell Neurosci. 2024 May 20;18:1390557. doi: 10.3389/fncel.2024.1390557 (PMC11145718; doi:10.3389/fncel.2024.1390557)
Supplement: Supplementary file 2 [file Image_1.pdf]

Supplementary Material

Odorant receptor co-receptors affect expression of tuning receptors in *Drosophila*

Teng Long[1†], Pratyajit Mohapatra[1†], Sydney Ballou[1] and Karen Menuz[1,2,3*]

[1]Department of Physiology and Neurobiology, University of Connecticut, Storrs, CT, USA

[2]Connecticut Institute for the Brain and Cognitive Sciences, University of Connecticut, Storrs, CT, USA

[3]Institute for Systems Genomics, University of Connecticut, Storrs, CT, USA

[†]These authors contributed equally to this work and share first authorship

***Correspondence:** karen.menuz@uconn.edu

Table S1. Fly lines used in this study

**Genotype**	**Figure(s)**
wCS; +; +	Figure 1, 4, 5, 6C-D, 7, S2, S3, S4
wCS; Ir25a^{2}; +	Figure 5, 7, S4
wCS, Ir8a^{1}; +; +	Figure 5, 6C-D, 7, S4
wCS; +; Orco^{2}	Figure 1, 4, 7, S2, S3
wCS; +; Ir76b^{1}	Figure 5, 7, S4
wCS; Ir25a^{2}; Ir76b^{1}	Figure 5, 7, S4
w; Or13a-GAL4/10XUAS-IVS-mCD8::RFP; Orco^{2}	Figure 2A-B, S3
w; Or13a-GAL4/10XUAS-IVS-mCD8::RFP; +	Figure 2A-B, S3
w; Or19a-GAL4/10XUAS-IVS-mCD8::RFP; Orco^{2}	Figure 2C-D, S3
w; Or19a-GAL4/10XUAS-IVS-mCD8::RFP; +	Figure 2C-D, S3
w; Or49b-GAL4/10XUAS-IVS-mCD8::RFP; Orco^{2}	Figure 2E-F, S3
w; Or49b-GAL4/10XUAS-IVS-mCD8::RFP; +	Figure 2E-F, S3
w; Or83c-GAL4/10XUAS-IVS-mCD8::RFP; Orco^{2}	Figure 2G-H, S3
w; Or83c-GAL4/10XUAS-IVS-mCD8::RFP; +	Figure 2G-H, S3
w; Or82a-GAL4/10XUAS-IVS-mCD8::RFP; Orco^{2}	Figure 2I-J, S3
w; Or82a-GAL4/10XUAS-IVS-mCD8::RFP; +	Figure 2I-J, S3
w; Or13a-GAL4/UAS-RedStinger, UAS-FLP.D, UBI-p63E(FRT.STOP)Stinger; Orco^{2}	Figure 3A-B
w; Or13a-GAL4/UAS-RedStinger, UAS-FLP.D, UBI-p63E(FRT.STOP)Stinger; +	Figure 3A-B
w; Or19a-GAL4/UAS-RedStinger, UAS-FLP.D, UBI-p63E(FRT.STOP)Stinger; Orco^{2}	Figure 3C-D
w; Or19a-GAL4/UAS-RedStinger, UAS-FLP.D, UBI-p63E(FRT.STOP)Stinger; +	Figure 3C-D
w; UAS-RedStinger, UAS-FLP.D, UBI-p63E(FRT.STOP)Stinger; +	Figure 3
wCS,Ir8a^{1}/Y; 10XUAS-IVS-mCD8::RFP/+; Ir75a-GAL4/+	Figure 6A-B
wCS/Y; 10XUAS-IVS-mCD8::RFP/+; Ir75a-GAL4/+	Figure 6A-B
wCS,Ir8a^{1}/Y; Ir64a-GAL4/UAS-RedStinger, UAS-FLP.D, UBI-p63E(FRT.STOP)Stinger; +/TM2	Figure 6E-F
wCS/Y; Ir64a-GAL4/UAS-RedStinger, UAS-FLP.D, UBI-p63E(FRT.STOP)Stinger; +/TM2	Figure 6E-F

Dataset S1. Antennal expression and differential gene expression in co-receptor mutants. The first spreadsheet “RPM” lists the reads per million mapped reads of each FlyBase Gene Symbol in each sample of the different genotypes and time points examined. The second spreadsheet “GLM” shows the results of the EdgeR generalized linear model differential gene expression analysis carried out with 7 DPE samples. The next five spreadsheets shows the results of the EdgeR ExactTests carried out on a pairwise basis between WT and different co-receptor mutant genotypes, with analysis for each mutant on a separate spreadsheet (“ExactTest Orco vs WT”, “ExactTest Ir8a vs WT”, “ExactTest Ir25a vs WT”, “ExactTest Ir76b vs WT”, and “ExactTest Ir25a;Ir76b vs WT”). The next spreadsheet “all DE genes” contains all identified DE genes (protein coding and non-protein coding) in each genotype with > 2-fold change compared to WT and FDR<0.01 in both the GLM and ExactTest analysis. The results of the ExactTest are shown. The final spreadsheet “206 protein encoding DE genes” categorizes the identified genes across genotypes and grouped by predicted molecular function. The first column lists the FlyBase Gene Symbol used in the previous spreadsheets, the second and third columns contain the updated FlyBase Gene Symbols and FlyBase Gene ID. Note that although CG30456 was analyzed in our analysis, it was recently split into two genes, CG46491 and CG46492. Columns D-H indicate whether the gene was identified as a DE gene in $Orco^{2}$, $Ir8a^{1}$, $Ir25a^{2}$, $Ir76b^{1}$, and/or $Ir25a^{2};Ir76b^{1}$ flies and whether it is up- or downregulated in that genotype. Columns I-O contain descriptors of the DE genes obtained from functional annotation with DAVID.

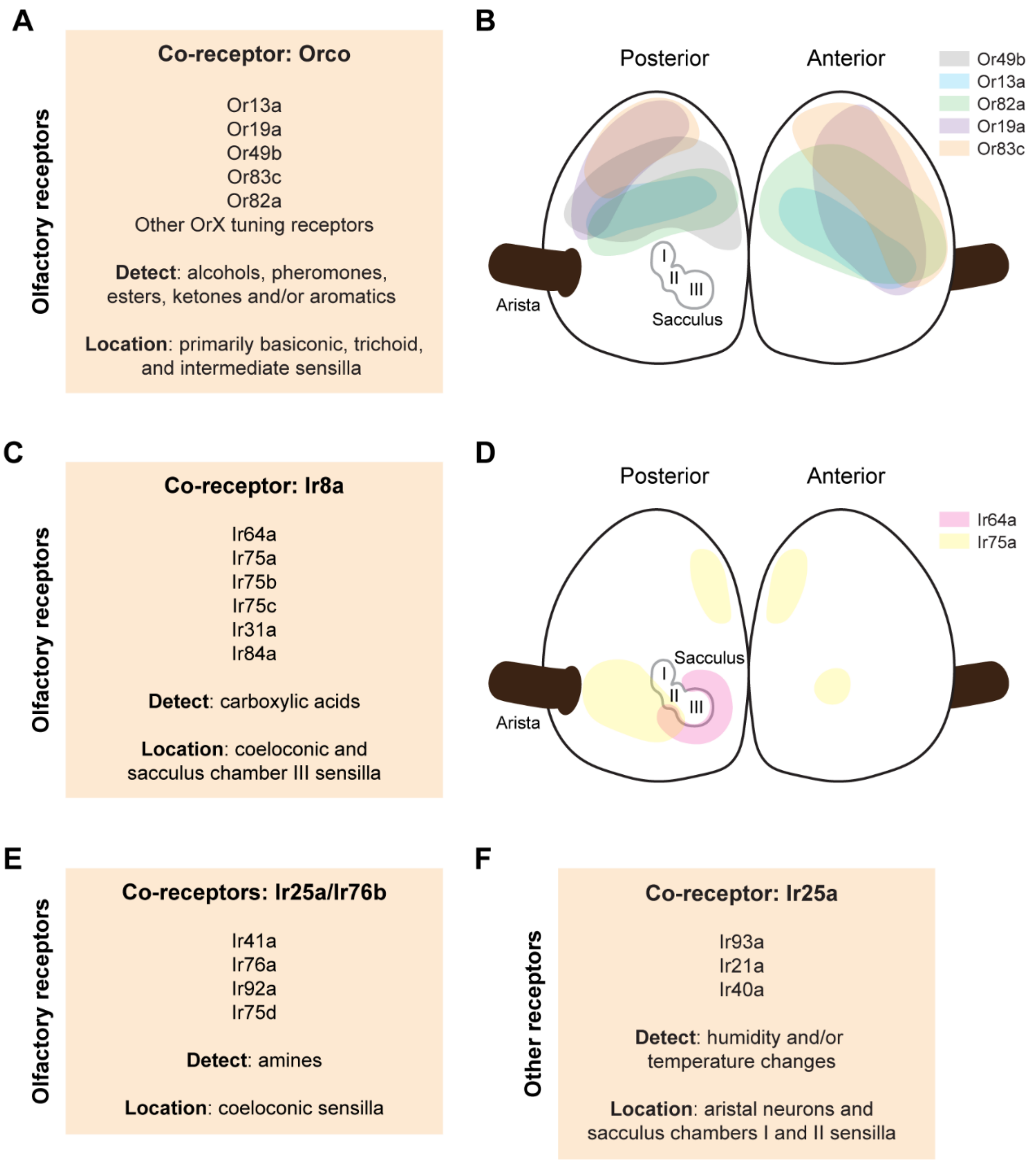

Figure S1. Chart of antennal IR and OR receptors analyzed in this study. (A) The chart lists the different Orco-dependent olfactory tuning receptors examined in this study. Their sensillar localization and typical odorant stimuli are also described. (B) A map showing the antennal localization of neurons labeled with OrX-GAL4 lines in this study. (C) The chart lists all Ir8a-dependent olfactory tuning receptors, their typical odorant stimuli, and sensillar localization. (D) A map showing the antennal localization of neurons labeled by either Ir64a-GAL4 or Ir75a-GAL4 in this study. (E) The chart lists all olfactory tuning receptors that are dependent on Ir25a and Ir76b for their function, their typical odorant stimuli, and sensillar localization. (F) Similar to (E), but for Ir25a-dependent antennal receptors that utilize IR family receptors for hygrosensation and thermosensation.

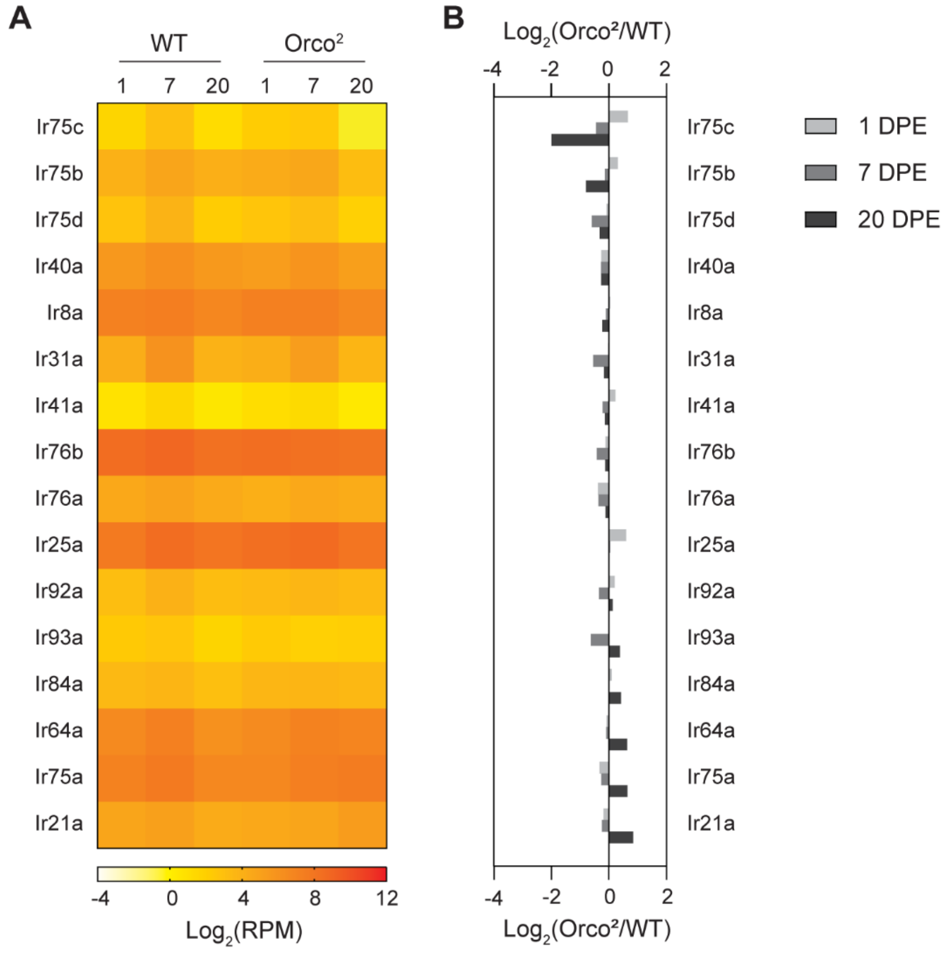

Figure S2. Expression of IR tuning receptors is unaffected in *Orco²* flies. (A) Heat map showing the average antennal expression in $\log_2$(RPM) for each of the detected antennal IR genes at 1, 7, and 20 DPE in WT and *Orco²* flies. (B) Bar graph reporting the $\log_2$ of the expression ratio between *Orco²* and WT flies for antennal-expressed members of the IR family. Antennal IRs are arranged in descending order of the $\log_2$ expression ratio at 20 DPE.

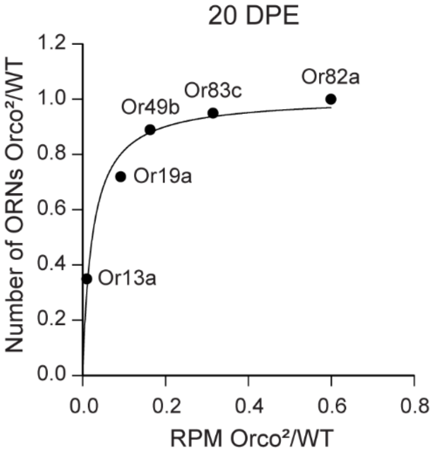

Figure S3. Relationship between RNASeq expression and apparent neuron loss in *Orco*^{2} flies. Graph comparing the relative level of OrX mRNA expression in *Orco*^{2} flies to WT controls versus the relative number of associated neurons in whole mounts from *OrX-GAL4>UAS-mCD8::RFP* flies, both at 20 DPE. Data fit well to a hyperbolic curve (R^2 0.96).

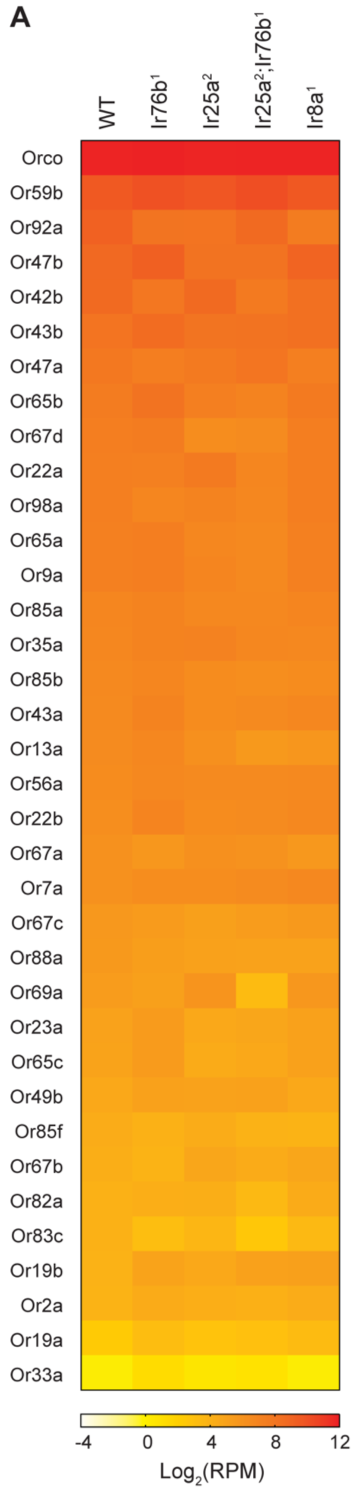

Figure S4. Expression of OR tuning receptors is unaffected in IR co-receptor mutants. (A) Heat map showing the average antennal expression in log_2(RPM) for known antennal OR genes in WT, *Ir76b*[1], *Ir25a*[2], *Ir25a*[2]*;Ir76b*[1], and *Ir8a*[1] flies. OR genes are arranged in descending order of expression in WT flies.
